# The validation of published utility mapping algorithms: an example of EORTC QLQ-C30 and EQ-5D in non-small cell lung cancer

**DOI:** 10.1186/s13561-020-00269-w

**Published:** 2020-04-21

**Authors:** Joanne Gregory, Matthew Dyer, Christopher Hoyle, Helen Mann, Anthony J. Hatswell

**Affiliations:** 1 BresMed Health Solutions, Steel City House, West Street, Sheffield, S1 2GQ UK; 2 AstraZeneca, 126-130 Hills Road, Cambridge, CB2 1RY UK; 3 Delta Hat, 212 Tamworth Road, Nottingham, NG10 3GS UK

**Keywords:** Utility mapping, EQ-5D, NSCLC, Quality of life

## Abstract

**Background:**

Mapping algorithms can be used to generate health state utilities when a preference-based instrument is not included in a clinical study. Our aim was to investigate the external validity of published mapping algorithms in non-small cell lung cancer (NSCLC) between the EORTC QLQ-C30 and EQ-5D instruments and to propose methodology for validating any mapping algorithms.

**Methods:**

We conducted a targeted literature review to identify published mappings, then applied these to data from the osimertinib clinical trial programme. Performance of the algorithms was evaluated using the mean absolute error, root mean squared error, and graphical techniques for the observed versus predicted EQ-5D utilities. These statistics were also calculated across the range of utility values (as well as ordinary least squares and quantile regression), to investigate how the mappings fitted across all values, not simply around the mean utility.

**Results:**

Three algorithms developed in NSCLC were identified. The algorithm based on response mapping (Young et al., 2015) fitted the validation dataset across the range of observed values with similar fit statistics to the original publication (overall MAE of 0.087 vs 0.134). The two algorithms based on beta-binomial models presented a poor fit to both the mean and distribution of utility values (MAE 0.176, 0.178).

**Conclusions:**

The validation of mapping algorithms is key to demonstrating their generalisability beyond the original dataset, particularly across the range of plausible utility values (not just the mean) – perceived patient similarity being insufficient. The identified algorithm from Young et al. performed well across the range of EORTC scores observed, and thus appears most suitable for use in other studies of NSCLC patients.

## Introduction

Health state utilities are used in economic evaluations of health care technologies and allow for the estimation of quality-adjusted life years (QALYs) gained by interventions. Their use is required for various health technology appraisal bodies, such as the National Institute for Health and Care Excellence (NICE), who prefer the use of the EQ-5D instrument [1]. Where the EQ-5D has not been collected in a clinical study, ‘mapping’ can be used to estimate the score that would have been recorded, which is conditional on the responses to questionnaires that were administered.

In recent years, guidance for best practice in mapping has proliferated, with established guidance on developing mapping algorithms [2], and for how these should be reported [3]. However, there exists no guidance for how the selection of a mapping algorithm should be conducted (particularly where several algorithms are available) and, where validation exercises are conducted, how these should be performed. This is particularly problematic as mappings between the same instruments have been shown to vary in their accuracy in other datasets [4, 5], with NICE Decision Support Unit recommendations being that as a result algorithms should be developed in patients with similar characteristics [6].

The number of mappings available between the EORTC QLQ-C30, a cancer-specific instrument, and the EuroQol EQ-5D was highlighted by Doble & Lorgelly (2015), who attempted to validate the published mappings in a study of 1727 Australian cancer patients that completed both instruments. This validation exercise (which included patients with all cancer types, and included sub-groups of cancer types) covered 10 mappings which were developed in a variety of populations (some single diseases, and some mixed populations) [4]. The result of this study demonstrated that two algorithms produced reasonable estimates of predicted utility (compared to observed utility), but that eight of the algorithms did not achieve this. This validation was later updated by Woodcock & Doble (2018) who applied five additional mapping algorithms. A similar pattern was seen by Crott et al., who found limited external validity of an algorithm, originally developed on a data set of patients with advanced breast cancer across external datasets in haematological cancers and NSCLC [7].

The objective of this study was to apply the approach of Doble & Lorgelly for validation of existing mappings to EGFR mutation-positive non-small cell lung cancer (NSCLC) instead of a mixed cohort of cancers. This involved identifying which lung cancer mappings applicable to UK value sets performed well in a second dataset of similar patients, and thus were most likely to have external validity to other datasets in NSCLC where only QLQ-C30 was measured. To do this, we had access to the AURA2 [NCT02094261] [8] and AURA3 [NCT02151981] [9] clinical studies, which collected both QLQ-C30 and EQ-5D-5 L questionnaires in previously treated EGFR and T790M mutation-positive NSCLC patients. In the process of performing this validation, we aimed to also identify issues surrounding the approach to validation of mapping algorithms.

## Methods

### Identification and selection of existing mapping algorithms

Three methods were used to identify published mappings algorithms. The first was a search of the Oxford mapping database v6.0 [10] for all mappings between the EORTC-QLQ-C30 and any version of the EQ-5D. The second method involved searching MEDLINE (via PubMed) using the search “mapping” AND “lung” AND “EQ-5D*”.

For the algorithms identified, we then selected those that were either based primarily on patients with lung cancer, or at least included patients with lung cancer. This criterion was added as it is not known whether mappings between the EORTC-QLQ-C30 and EQ-5D perform similarly in patients with different cancer types, whilst in other instruments the relationships between scales has been shown to vary when the cause of the symptoms is not identical [5] – it is not known whether this finding holds between cancers, though given the different symptoms patients can face we felt it conservative to restrict our analysis to papers including a ‘similar’ group. Such differences between groups were also posited as a potential reason why the algorithm tested in Crott et al. did not perform well in external datasets from different cancers [7]. For this reason, we only included mappings which included a meaningful (defined as n > 30) number of patients with lung cancer – with the justification that had the mapping not performed well in the group, this would be identified by the original study authors.

### Validation datasets

AURA2 is a phase II single arm trial of osimertinib which included 210 patients. AURA3 is a phase III randomised two-arm trial of osimertinib versus platinum–pemetrexed which included 419 patients. A total of 4395 EQ-5D-3 L and 4626 QLQ-C30 observations were collected from AURA2 and AURA3 combined. These were matched on date and visit to obtain a total of 4382 utility observations from 594 patients for validation of the existing algorithms. Observed EQ-5D-3 L utility values (UK tariff) were derived using the cross-walk algorithm [11] from the EQ-5D-5 L observed responses in the AURA trials.

Mapping algorithms obtained from the literature review were then applied to the QLQ-C30 data to obtain predicted EQ-5D-3 L utility values (due to our inclusion criteria, all of these used the UK tariff). Where multiple mapping algorithms were provided in one publication, the author’s preferred mapping was utilised (as in Doble & Lorgelly). EQ-5D and QLQ-C30 questionnaires completed on the same day were matched to produce pairs of observed and mapped utility observations. All analysis was performed using SAS statistical software (SAS Institute, Cary, NC, USA) and R (R Core Team (2019)).

### Validation approach

**Fig. 1:**
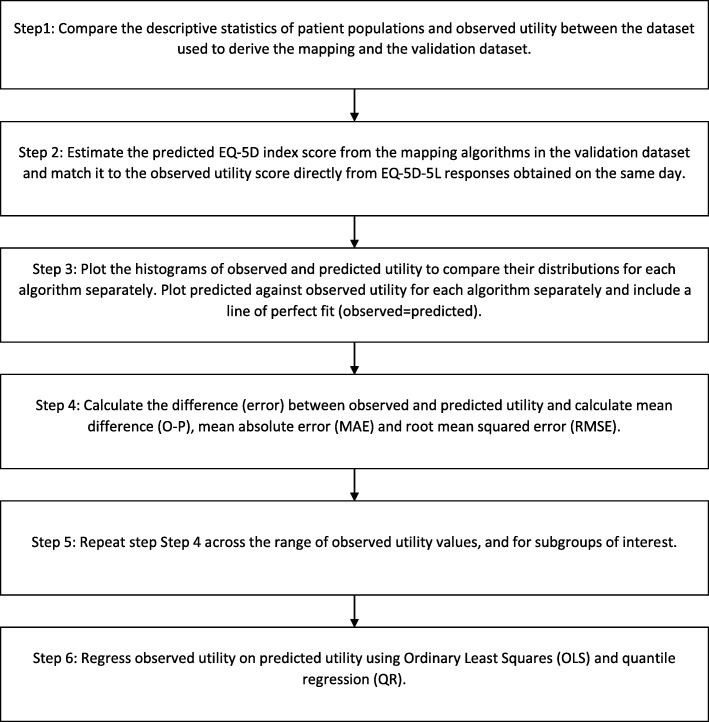
Steps for assessing external validity of mapping algorithms

External validity was assessed in steps summarised in Fig. 1, and described in detail below.

### Comparison of populations

Descriptive statistics of patient populations and observed utility values (including mean, standard deviation and range) of all observed EQ-5D utility values were compared across AURA2, AURA3, and published studies to gauge the amount of overlap.

### Approach to assessing mappings in the overall dataset

After applying the mappings and matching the observed and mapped observations, graphical methods including histograms and scatterplots were used to show the difference between observed and predicted (mapped) utility. Goodness of fit statistics were calculated between the observed and predicted data. In line with Doble & Lorgelly, we calculated the mean absolute error (MAE), root mean squared error (RMSE) and the mean observed minus mean predicted utility (O-P) value. The MAE is the absolute value of any error in prediction, whilst the RMSE gives an increasing penalty as the prediction errors increase. Low values for MAE and RMSE suggest the algorithm is precise in fitting to the data, whereas a difference between observed and predicted utilities measures the level of bias (i.e. systematic over- or under-prediction of utility).

### Approach to assessing mapping across the range of plausible utility values

When considering applying a mapping to a different dataset, arguably more important than the overall fit is that the fit should be reasonable across the range of plausible utility values. This is important because mapping algorithms may fit well to the mean utility of the observed data but not to the extremities [2]. This is particularly problematic if the dataset to be used in mapping has a different mean utility (or range of utilities) to the dataset that the algorithm was derived in; as the algorithm is unlikely to fit to the majority of the data. This could introduce bias in any subsequent analysis, and may not be detected as it is likely that directly measured utilities are not available (else the mapping would not be used).

To assess this fit across the range of plausible utilities, the goodness of fit assessment was repeated across the observed EQ-5D range (EQ-5D < 0.5, 0.5 < =EQ-5D < 0.75, 0.75 < =EQ-5D < 1), being mindful of the number of observations available to create comparisons. In addition, observed EQ-5D utility values were regressed on predicted values using ordinary least squares (OLS) and quantile regression (QR), where the quantiles are specified as 10%, 25%, 50%, 75%, and 90%. For OLS, a coefficient (gradient of the slope) close to 1 together with an intercept of 0 indicates that observed utility is a good fit to predicted utility. The QR shows how this coefficient changes over the quartiles of the observed utility value.

### Approach to assessing mapping across subgroups

To ensure any mapping fits well under different circumstances, we also compared the fit of the mapping algorithm to different subgroups. Subgroup analyses were explored because there are likely to be imbalances between the number of pre/post progression observations between studies, as well as differences in baseline characteristics between mapping, validation, and any other dataset to which the mapping is applied. Subgroups considered in this analysis were health state at time of questionnaire (pre/post progression), trial (AURA2, AURA3), gender (male, female) and age at screening (< 65, ≥65).

## Results

### Identification and selection of existing mapping algorithms

The literature search covered all publications to 17 July 2018. Seven publications were identified, of which three met the inclusion criteria of mappings between the QLQ-C30 and EQ-5D derived using lung cancer patients and scored using UK tariffs (Young et al. 2015, Khan & Morris 2014, and Khan et al. 2016) [12–14]. The PRISMA diagram is presented in the electronic supplementary material.

The mapping by Young et al. utilised 771 patients with all types of cancer (12.8% of whom had lung cancer) from Canada. A number of different types of statistical models were tested (including OLS regression, Tobit, two-part models, splining, and response mapping); however, response mapping was found to be the authors’ preferred mapping algorithm. Rather than predicting the EQ-5D index score for a particular tariff, response mapping predicts the probability of a patient scoring 1, 2, or 3 for each of the five EQ-5D-3 L dimensions using multinomial logistic regression models. The mapping was first reported in a UK Health Technology Assessment report [15], and subsequently used in a NICE Decision Support Unit (DSU) report [6].

The mappings by Khan & Morris and Khan et al. utilised 670 and 98 patients, respectively, with NSCLC from the UK. Both papers tested different types of statistical models (including linear mixed effects, Tobit, quantile, quadratic, censored least absolute deviation (CLAD) and beta-binomial models). In both cases, the beta-binomial model was found to be the authors’ preferred mapping algorithm. The beta-binomial model assumes a scale between 0 and 1 and can model responses that are unimodal or binomial, with varying levels of skewness. It can be used to predict EQ-5D using a standard logit link function such that predicted utility (P) = exp. (Xβ)/[1 + exp. (Xβ)].

### Comparison of populations

Baseline characteristics and mean utility values from the three mapping papers and the AURA2 and AURA3 studies show that the patients in AURA2 and AURA3 (the validation dataset) appear to be younger. In addition, there appear to be more female patients, and a better health state utility than the patients used to derive any of the mapping algorithms (Table 1).

**Table 1:** Baseline characteristics of each study compared to the validation study

Study	Number of patients	Mean age	% Male	Stage of disease	Utility - Mean (SD) [range]
AURA 2	203	63.0	31.0	I/II 10% III 14% IV 75%	0.789 (0.219) [− 0.594 to 1]
AURA 3	391	61.4	36.3	I/II 10% III 10% IV 80%	0.808 (0.209) [−0.594 to 1]
AURA2/AURA3 combined	594	61.9	34.5	I/II 10% III 11% IV 78%	0.799 (0.214 [−0.594 to 1]
Young et al. (2015)	771	68.3	44.1	NR	0.58 (0.342) [− 0.594 to 1]
Khan and Morris (2014)	670	77	NR	I/II 0% III/IV 100%	0.61 (0.29) [−0.043 to 1]
Khan et al. (2016)	98	69	44	I/II 27% III 32% IV 38%	0.515 (0.308) [−0.594 to 1]

### Assessment of mappings in the overall dataset

**Fig. 2:**
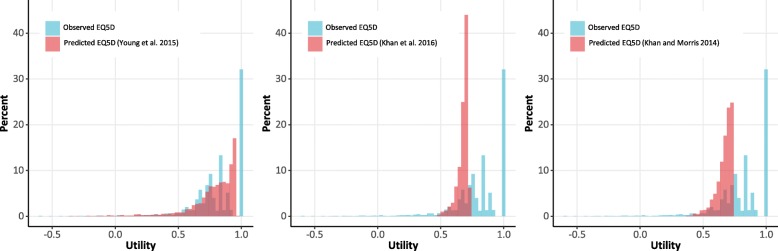
Histograms of observed and mapped utility

The distribution of predicted EQ-5D utility values from the three mapping algorithms overlaid on top of the observed EQ-5D utility values for the combined AURA2 and AURA3 dataset show that the Young et al. values are relatively similar to the observed data in terms of the overall shape of the data. As it is not possible to predict a utility of 1 due to the type of algorithm used, this large peak in the observed utility was not exactly matched in predicted utility; however, there is a large peak in predicted utility at 0.90–0.95, which may reflect this. The predicted utilities from the other two algorithms do not capture the distribution of the observed utilities, which resulted in a much smaller range of predicted values compared to the observed values (Fig. 2).

**Fig. 3:**
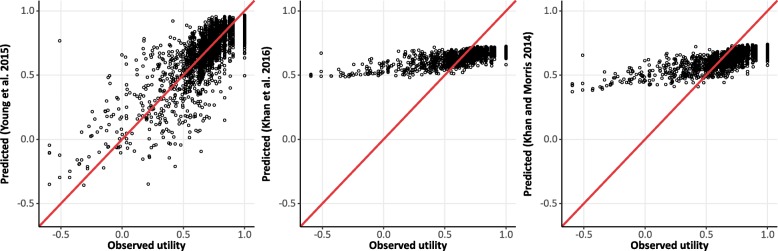
Scatter plots of predicted vs observed utility

A scatterplot of observed utility against predicted utility from the three mapping algorithms (including a line of perfect fit where observed = predicted) shows that the predicted utilities from the Young et al. algorithm fitted the observed data relatively well, with an equal spread above and below the line of perfect fit, suggesting that overall, the mapping neither over- or under-predicts utility. However, for both beta-binomial algorithms (Khan and Morris and Khan et al.), the plots show that the mappings do not fit the observed data, which is mainly due to the small range of utility values produced by both algorithms (Fig. 3). Although there is a positive correlation between observed and predicted utility for each of the two mappings, the gradients do not match the line of perfect fit.

These patterns are quantified in the numerical summaries of the three mapping algorithms including mean observed utility minus mean predicted utility (O-P), MAE and RMSE calculated in the validation dataset, which are compared to the values reported in the original publications. Again, the mapping by Young et al. provides a good fit to the observed data since the values are relatively small. For this algorithm, the MAE is smaller (0.087) than the original dataset (0.134) and the validation by Woodcock & Doble (0.096) [16]. Furthermore, the RMSE and O-P values are also reasonably low. On the other hand, the two beta-binomial mappings do not perform as well in the validation dataset as shown by the higher values compared to applying the Young et al. algorithm, despite low O-P and MAE values reported in the original paper, i.e. the mappings appear to have been a good fit to the data on which they were based on, but not to the dataset we had available (Table 2). This poor fit is also reflected in the validation by Woodcock & Doble where MAE values for the beta-binomial model by Khan and Morris [13] was higher (0.212) than both the validation reported here (0.176) and in the original dataset (0.10).

**Table 2:** Observed vs predicted goodness of fit statistics

Mapping	Source of utility data	N	Observed mean (95% CI)	Predicted mean (95% CI)	O-P	Mean absolute error (MAE)	Root mean squared error (RMSE)
Young et al. (2015) [12]	Taken from the source paper	771	0.579 (0.555, 0.603)	0.573 (0.552, 0.594)	0.007	0.134	NR
	Mapped to AURA data via ‘crosswalk’	4382	0.799 (0.793, 0.805)	0.777 (0.771, 0.783)	0.022	0.087	0.119
Khan and Morris (2014) [13]	Taken from the source paper	2038	0.610 (0.597, 0.623)	0.608 (0.600, 0.616)	0.002	0.10	0.09
	Mapped to AURA data via ‘crosswalk’	4382	0.799 (0.793, 0.805)	0.67 (0.668, 0.672)	0.129	0.176	0.211
Khan et al. (2016) [14]	Taken from the source paper	985	0.515 (0.496, 0.534)	0.518 (0.507, 0.529)	−0.003	0.099	0.113
	Mapped to AURA data via ‘crosswalk’	4382	0.799 (0.793, 0.805)	0.677 (0.676, 0.678)	0.122	0.178	0.219

Key: *N* Number of questionnaires completed, *NR* Not reported, *O-P* Observed mean utility minus predicted mean utility

### Assessment of mappings across the range of plausible utility values

The numerical summaries are also calculated across the range of observed EQ-5D values. For the Young et al. mapping, both the MAE and the RMSE are smallest for higher values of observed utility and are considerably higher for the lower observed utility values, showing that the mapping does not fit as well for low values. For both the beta-binomial models, the mapping fits the observed data very well between 0.5 and 0.75, as seen by the low MAE and RMSE. However, overall and for the high and low values, the fit statistics are much worse, representing a poor fit across the wider range of observed values (Table 3).

**Table 3:** Observed vs predicted goodness of fit statistics over the range of observed EQ-5D values

Mapping	Overall ***N*** = 4382			EQ-5D < =0.5 ***N*** = 288			0.5 < EQ-5D < =0.75 ***N*** = 1147			0.75 < EQ-5D < =1 ***N*** = 2947		
	O-P	MAE	RSME	O-P	MAE	RSME	O-P	MAE	RSME	O-P	MAE	RSME
Young et al. (2015) [12]	0.022	0.087	0.119	−0.092	0.207	0.264	− 0.007	0.090	0.123	0.045	0.074	0.091
Khan and Morris (2014) [13]	0.129	0.176	0.211	−0.312	0.313	0.384	0.038	0.058	0.069	0.208	0.208	0.223
Khan et al. (2016) [14]	0.122	0.178	0.219	−0.360	0.360	0.427	0.013	0.046	0.057	0.212	0.212	0.229

Key: *MAE* Mean absolute error, *N* Number of questionnaires completed, *O-P* Difference between mean observed and predicted EQ-5D utility values, *RSME* Root mean square error

**Table 4:** Observed regressed on predicted utility using OLS and QR

Mapping	OLS		QR (10%)		QR (25%)		QR (50%)		QR (75%)		QR (90%)	
	Intercept	Coefficient: predicted value	Intercept	Coefficient: predicted value	Intercept	Coefficient: predicted value	Intercept	Coefficient: predicted value	Intercept	Coefficient: predicted value	Intercept	Coefficient: predicted value
Young et al. (2015) [12]	0.071	0.938	−0.270	1.209	−0.099	1.069	−0.022	1.076	0.214	0.842	0.444	0.619
Khan and Morris (2014) [13]	−1.914	2.915	−1.900	3.822	−1.438	3.241	−1.146	2.924	−0.724	2.384	−0.366	1.924
Khan et al. (2016) [14]	−1.153	4.009	−2.844	5.155	−2.163	4.266	−2.073	4.277	−1.351	3.295	−0.681	2.397

Key: *OLS* Ordinary least squares, *QR* Quantile regression

This pattern is explored using OLS and QR for observed utility regressed on predicted utility for each of the three mapping algorithms separately. The coefficient for Young et al. is close to 1 and the intercept is close to 0, suggesting that the mapping is a good fit to the observed data throughout the range. As expected from the plots and numerical summaries over the range of EQ-5D, the QR coefficient is closer to 1 at the median: Q (0.5) compared to the first and last 10% of the data. This is comparable to the variation seen by many of the algorithms validated in Doble & Lorgelly and Woodcock & Doble. The coefficients from beta-binomial mappings are not close to 1, providing further evidence that they do not fit well in the validation dataset (Table 4).

### Assessment of mappings across subgroups

Results were consistent across subgroups including heath state (progression status) at time of questionnaire, trial, gender, and age at screening. Goodness-of-fit statistics by group are presented in the electronic supplementary material (Supplementary Table 1).

## Discussion

The results of our study show that not all mappings have validity to be used outside of the dataset used to originally produce the mapping. Whilst the response mapping by Young et al. gave similar goodness-of- fit statistics in the AURA2 and AURA3 studies as in the original publication, the studies by Khan & Morris and Khan et al. showed a poor fit to the data, particularly when considering values beyond the mean utility. Simply comparing the populations in the original studies to those in our validation datasets would therefore seem an insufficient step for determining the optimal mapping to use - and highlights the importance of validating published mapping algorithms in external datasets.

We believe the poor fit across the range of possible values to be particularly concerning. Mapping algorithms that fit the mean of the data but not the range are unlikely to generalise well to datasets with different patient characteristics (and thus a higher or lower mean utility) – as seen in Table 1, the range of mean utilities in the studies ranges from 0.515 in Khan et al. 2016 to 0.799 in our validation dataset. As mapping algorithms are used in the absence of directly measured utility, poor fit across different ranges could bias decisions using mapped utilities as inputs, with the problem going undetected. For this reason, we would thus be extremely cautious about using utilities derived from the Khan & Morris or Khan et al. studies for decision making.

The statistical methods used to derive these mappings could contribute to the fitting of the model to external data. The predicted utilities in Young et al. were derived using a response mapping technique that models the probability of a patient scoring 1, 2, or 3 for each of the five EQ-5D-3 L dimensions, which are then used to calculate the EQ-5D index score rather than predicting the EQ-5D index score directly (as is done in beta-binomial methods in the other two papers). Although this type of mapping is uncommon, it appears to perform well in our validation exercise [10]. In addition, these methods allow for prediction of utility using any country tariff by scoring the predicted responses using the appropriate coefficients. Therefore, it may be beneficial for future work deriving mapping algorithms to consider these more complex methods alongside other statistical models such as limited dependent mixture models and ordinary least squares – the final chosen methods are likely to depend on both statistical fit, and the number of observations available (response mapping has greater data requirements).

When conducting the work, we identified several issues that are yet to be resolved on how best to validate mapping algorithms. Most notable was selection of the most appropriate goodness of fit statistic(s) and defining the values of these statistics that represent a ‘good’ fit. Selective reporting across papers complicates this issue as it is then not always possible to compare mapping algorithms on the same metrics. For this reason, we calculated all statistics reported in any of the original papers to make comparisons between the goodness of fit in the original paper and the corresponding values in our validation exercise. We would recommend this approach in addition to the steps proposed by Doble & Lorgelly, until such guidelines exist for the validation of mapping studies – it is then at least possible to see whether the mapping algorithm fits as well as in the original paper and thus may be more likely to generalise to other datasets.

In addition to the limitation around goodness of fit statistics, there is also the possibility that algorithms have been incorrectly applied in the case of the beta-binomial models. Despite following the methods described in the papers to derive the predicted utility (and attempting to contact the original authors), it is possible that these algorithms have not been applied as intended, especially for the two beta-binomial algorithms where the fit to our data is poor, though this was also the case with the Cancer 2015 data reported in Woodcock & Doble. This highlights a disadvantage of applying non-standard methods (such as beta-binomial), as application errors are more likely to occur due to the difficulty in explicitly reporting the algorithms in sufficient detail (or with rounding errors impacting calculations). This limitation (in part) could be avoided if future mapping publications provide worked examples in electronic form, thus reducing the chance of improper application and eliminating the need to manually enter covariates with the chance of data entry errors.

We are also conscious that a limitation of our analysis is that the EQ-5D data available from the AURA trial programme was the 5 L version of the questionnaire which is then used in the crosswalk to the EQ-5D-3 L version and this introduces an additional (unavoidable) level of uncertainty. However, this does reflect the state of the literature, with many published mappings using the EQ-5D-3 L, whilst an increasing number of studies use the (newer) EQ-5D-5 L. This is worth noting as the emerging literature suggests the 5 level version of the instrument behaves differently to the 3 L version [17] – so much so, that NICE in the UK have stated a preference to use the 3 L version in HTA submissions until these differences are resolved [18].

Through this work, we also believe there exists a need for further research in two key areas. The first of these is regarding whether mapping algorithms are specific to the conditions in which they are derived, particularly in the case of the QLQ-C30, which has been designed for all cancers (and which have a wide range of possible severities and symptoms). Using steps similar to those in this study may allow the cross validation of mappings from different cancers, with the ultimate aim of understanding where derived mappings are similar, and where they are not. The second main area of research we believe is required surrounds the approach to the validation of mappings. The mixed picture that exists in the literature surrounding the external validity of mappings is concerning, with a standardised approach to validation likely to increase confidence in claims. A further area for research, though more conceptual in nature, regards how different mappings can be synthesized – at present, the main issue is choosing which mapping to use, as opposed to recognising that each may provide information (as is the case when meta-analysing clinical studies).

## Conclusion

This exercise demonstrates that the mapping by Young et al. fits well to our validation dataset, as it also did to the Cancer 2015 data available to Doble & Lorgelly (who listed it as a well performing algorithm). Whilst this result does not guarantee it will always represent a good fit to all data, should mapping be required to derive utility values for use in economic evaluations of technologies in NSCLC then the algorithm derived by Young et al. would be our preferred approach. In performing this validation, we also identified a number of issues around the validation of datasets, highlighting that this is an important process prior to their use in economic evaluations.

## Supplementary information

**Additional file1: Figure S1.** PRISMA diagram for the literature search. **Table S1.** Observed vs predicted goodness of fit statistics over subgroups.

## Data Availability

Published algorithms are applied to confidential patient data.

## Abbreviations

EORTC QLQ-C30: European Organization for Research and Treatment of Cancer Quality of Life Questionnaire 30 item
EQ-5D: EuroQol 5 Dimension
MAE: Mean Absolute Error
NICE: National Institute for Health and Care Excellence
NSCLC: Non-small Cell Lung Cancer
OLS: Ordinary Least Squares
O-P: Observed minus Predicted
QALY: Quality-Adjusted Life Year
QR: Quantile Regression
RMSE: Root Mean Squared Error

## References

[1] NICE. Guide to the methods of technology appraisal 2013. NICE; 2013. Available from: https://www.nice.org.uk/process/pmg9/chapter/foreword. 27905712

[2] Longworth L, Rowen D (2013). Mapping to obtain EQ-5D utility values for use in NICE health technology assessments. Value Health.

[3] Petrou S, Rivero-Arias O, Dakin H, Longworth L, Oppe M, Froud R (2016). Preferred reporting items for studies mapping onto preference-based outcome measures: the MAPS statement. Qual Life Res Int J Qual Life Asp Treat Care Rehab.

[4] Doble B, Lorgelly P (2016). Mapping the EORTC QLQ-C30 onto the EQ-5D-3L: assessing the external validity of existing mapping algorithms. Qual Life Res Int J Qual Life Asp Treat Care Rehab.

[5] Hatswell AJ, Vegter S. Measuring quality of life in opioid-induced constipation: mapping EQ-5D-3 L and PAC-QOL. Heal Econ Rev. 2016;6 [Cited 2018 Mar 28]. Available from: https://www.ncbi.nlm.nih.gov/pmc/articles/PMC4839018/. 10.1186/s13561-016-0091-9 PMC4839018 27098897

[6] Longworth L, Rowen D. NICE DSU technical support document 10: the use of mapping methods to estimate health state utility values. Lond NICE. 2011; [Cited 2014 Nov 13]; Available from: http://www.nicedsu.org.uk/TSD%2010%20mapping%20FINAL.pdf. 28481491

[7] Crott R, Versteegh M, Uyl-de-Groot C (2013). An assessment of the external validity of mapping QLQ-C30 to EQ-5D preferences. Qual Life Res.

[8] Goss G, Tsai C-M, Shepherd FA, Bazhenova L, Lee JS, Chang G-C (2016). Osimertinib for pretreated EGFR Thr790Met-positive advanced non-small-cell lung cancer (AURA2): a multicentre, open-label, single-arm, phase 2 study. Lancet Oncol.

[9] Mok TS, Wu Y-L, Ahn M-J, Garassino MC, Kim HR, Ramalingam SS (2017). Osimertinib or platinum–Pemetrexed in EGFR T790M–positive lung Cancer. N Engl J Med.

[10] Dakin H, Abel L, Burns R, Yang Y (2018). Review and critical appraisal of studies mapping from quality of life or clinical measures to EQ-5D: an online database and application of the MAPS statement. Health Qual Life Outcomes.

[11] van Hout B, Janssen MF, Feng Y-S, Kohlmann T, Busschbach J, Golicki D (2012). Interim scoring for the EQ-5D-5L: mapping the EQ-5D-5L to EQ-5D-3L value sets. Value Health J Int Soc Pharmacoeconomics Outcomes Res.

[12] Young TA, Mukuria C, Rowen D, Brazier JE, Longworth L (2015). Mapping functions in health-related quality of life. Med Decis Mak.

[13] Khan I, Morris S. A non-linear beta-binomial regression model for mapping EORTC QLQ- C30 to the EQ-5D-3L in lung cancer patients: a comparison with existing approaches. Health Qual Life Outcomes. 2014; [Cited 2018 Mar 2];12. Available from: https://www.ncbi.nlm.nih.gov/pmc/articles/PMC4234877/. 10.1186/s12955-014-0163-7 PMC4234877 25388439

[14] Khan I, Morris S, Pashayan N, Matata B, Bashir Z, Maguirre J. Comparing the mapping between EQ-5D-5L, EQ-5D-3L and the EORTC-QLQ-C30 in non-small cell lung cancer patients. Health Qual Life Outcomes. 2016;14 [Cited 2018 Mar 2]. Available from: http://hqlo.biomedcentral.com/articles/10.1186/s12955-016-0455-1. 10.1186/s12955-016-0455-1 PMC4830017 27072351

[15] Longworth L, Yang Y, Young T, Mulhern B, Hernández Alava M, Mukuria C (2014). Use of generic and condition-specific measures of health-related quality of life in NICE decision-making: a systematic review, statistical modelling and survey. Health Technol Assess Winch Engl.

[16] Woodcock F, Doble B, Fox SB, Collins I, Hayes T, Singh M (2018). Mapping the EORTC-QLQ-C30 to the EQ-5D-3L: An Assessment of Existing and Newly Developed Algorithms. Med Decis Mak.

[17] Pennington B, Hernandez-Alava M, Pudney S, Wailoo A (2018). The impact of moving from EQ-5D-3L to -5L in NICE technology appraisals. Pharmaco Econ.

[18] National Institute for Health and Care Excellence. Position statement on use of the EQ-5D-5L valuation set. [Cited 2019 Dec 12]. Available from: https://www.nice.org.uk/Media/Default/About/what-we-do/NICE-guidance/NICE-technology-appraisal-guidance/eq5d5l_nice_position_statement.pdf.

