# Supplementary material for: The validation of published utility mapping algorithms: an example of EORTC QLQ-C30 and EQ-5D in non-small cell lung cancer
Source: Health Econ Rev. 2020 Apr 21;10:10. doi: 10.1186/s13561-020-00269-w (PMC7175479; doi:10.1186/s13561-020-00269-w)
Supplement: Supplementary file 1 — Additional file1: Figure S1. PRISMA diagram for the literature search. Table S1. Observed vs predicted goodness of fit statistics over subgroups. [file 13561_2020_269_MOESM1_ESM.docx]

Supplementary material

**Figure 1:** PRISMA diagram for the literature search

PubMed
(n = 7)

## Screening

## Included

## Eligibility

## Identification

Oxford mapping database
(n = 6)

Records after duplicates removed
(n = 7)

Records screened
(n = 7)

Records excluded
(n = 3)

2 developed using non-UK data

1 did not include EQ-5D

1

1 Developed

Full-text articles excluded (n = 1)
1 developed in breast cancer

Studies included in analysis
(n = 3)

Doble & Lorgelly 2015
(n = 2)

Full-text articles assessed for eligibility
(n = 4)

**Table 1:** Observed vs predicted goodness of fit statistics over subgroups

| Covariate | Level | N | Young et al. (2015) | | | Khan and Morris (2014) | | | Khan et al. (2016) | | |
| --- | --- | --- | --- | --- | --- | --- | --- | --- | --- | --- | --- |
|  |  |  | O-P | MAE | RMSE | O-P | MAE | RMSE | O-P | MAE | RMSE |
| Overall | | 4,382 | 0.022 | 0.087 | 0.119 | 0.129 | 0.176 | 0.211 | 0.123 | 0.178 | 0.219 |
| Health state | Pre-progression | 3,281 | 0.023 | 0.085 | 0.114 | 0.140 | 0.177 | 0.208 | 0.135 | 0.180 | 0.215 |
|  | Post-progression ^a^ | 4,254 | 0.017 | 0.090 | 0.133 | 0.098 | 0.171 | 0.217 | 0.085 | 0.172 | 0.227 |
| Trial | AURA2 | 2,042 | 0.020 | 0.087 | 0.117 | 0.123 | 0.173 | 0.209 | 0.114 | 0.175 | 0.217 |
|  | AURA3 | 2,340 | 0.024 | 0.087 | 0.121 | 0.135 | 0.178 | 0.212 | 0.130 | 0.181 | 0.221 |
| Gender | Male | 1,522 | 0.029 | 0.084 | 0.110 | 0.153 | 0.182 | 0.212 | 0.148 | 0.185 | 0.221 |
|  | Female | 2,860 | 0.018 | 0.088 | 0.123 | 0.117 | 0.172 | 0.210 | 0.109 | 0.175 | 0.218 |
| Age | < 65 years | 2,396 | 0.021 | 0.085 | 0.118 | 0.132 | 0.181 | 0.218 | 0.123 | 0.183 | 0.227 |
|  | >= 65 years | 1,986 | 0.023 | 0.089 | 0.120 | 0.127 | 0.169 | 0.201 | 0.122 | 0.173 | 0.209 |
| **Key:** MAE, mean absolute error; N, number of questionnaires completed; O-P, observed mean utility minus predicted mean utility; RMSE, root mean square error.  ^a^ Observations after a censored progression have been removed for this analysis. | | | | | | | | | | | |
